# A study on the treatment effects of *Crataegus pinnatifida* polysaccharide on non-alcoholic fatty liver in mice by modulating gut microbiota

**DOI:** 10.3389/fvets.2024.1383801

**Published:** 2024-03-27

**Authors:** Ping Hao, Xiaonan Yang, Wen Yin, Xinyi Wang, Yun Ling, Mengyao Zhu, Yue Yu, Shouhai Chen, Yuan Yuan, Xiaoyu Quan, Zhiheng Xu, Jiahui Zhang, Wenjia Zhao, Ying Zhang, Chunlian Song, Qing Xu, Shuangshuang Qin, Yi Wu, Xianghua Shu, Kunhua Wei

**Affiliations:** ^1^Ministry of Education (MOE) Joint International Research Laboratory of Animal Health and Food Safety, College of Veterinary Medicine, Nanjing Agricultural University, Nanjing, China; ^2^Key Laboratory of State Administration of Traditional Chinese Medicine for Production and Development of Cantonese Medicinal Materials/Guangdong Engineering Research Center of Good Agricultural Practice and Comprehensive Development for Cantonese Medicinal Materials, School of Chinese Materia Medica, Guangdong Pharmaceutical University, Guangzhou, China; ^3^National Engineering Research Center for Southwest Endangered Medicinal Resources Development, Guangxi Key Laboratory of Medicinal Resources Protection and Genetic Improvement, Guangxi Botanical Garden of Medicinal Plants, Nanning, China; ^4^College of Medicine (Institute of Translational Medicine), Yangzhou University, Yangzhou, China; ^5^Department of Chemistry, College of Sciences, Nanjing Agricultural University, Nanjing, China; ^6^College of Veterinary Medicine, Yunnan Agricultural University, Kunming, China; ^7^Institute of Biology, Guizhou Academy of Sciences, Guiyang, China

**Keywords:** *Crataegus pinnatifida*, polysaccharide, HepG2 cells, non-alcoholic fatty liver, gut microbiota

## Abstract

The objective of this study was to investigate the protective effect of *Crataegus pinnatifida* polysaccharide (CPP) on non-alcoholic fatty liver disease (NAFLD) induced by a high-fat diet (HFD) in mice. The findings demonstrated that CPP improved free fatty acid (FFA)-induced lipid accumulation in HepG2 cells and effectively reduced liver steatosis and epididymal fat weight in NAFLD mice, as well as decreased serum levels of TG, TC, AST, ALT, and LDL-C. Furthermore, CPP exhibited inhibitory effects on the expression of fatty acid synthesis genes FASN and ACC while activating the expression of fatty acid oxidation genes CPT1A and PPARα. Additionally, CPP reversed disturbances in intestinal microbiota composition caused by HFD consumption. CPP decreased the firmicutes/Bacteroidetes ratio, increased Akkermansia abundance, and elevated levels of total short-chain fatty acid (SCFA) content specifically butyric acid and acetic acid. Our results concluded that CPP may intervene in the development of NAFLD by regulating of intes-tinal microbiota imbalance and SCFAs production. Our study highlights that CPP has a potential to modulate lipid-related pathways via alterations to gut microbiome composition thereby ex-erting inhibitory effects on obesity and NAFLD development.

## 1 Introduction

Non-alcoholic fatty liver disease (NAFLD) is the primary cause of global chronic liver disease with fast-rising incidences (1). NAFLD is a metabolic syndrome in the liver with close relationship to obesity caused by metabolic changes on molecular pathological pathways, such as insulin resistance and impaired lipid metabolism (2–4). Since NAFLD is heavily influenced by factors such as high caloric intake and a lack of physical activity (5), changes of lifestyles such as diet adjustments and appropriate physical activities are the recommended treatment for NAFLD (6, 7). However, the lack of approved effective drugs for NAFLD indicates that discovering and identifying drug candidates to inhibit NAFLD has significant practical implications for clinical research (8). The liver is the organ most closely linked to the gut, and it is exposed to numerous bacteria and their metabolites. Microbiome alterations are thereby linked to many liver disorders, such as alcoholic liver disease, non-alcoholic liver disease, and primary sclerosing cholangitis (9). Our current research indicates that a high-fat diet (HFD) can induce dysbiosis of the intestinal microbiota, which contributes to the pathogenesis and progression of NAFLD through various mechanisms (10). There are growing evidences that disturbances in the gut microbiome play a key role in obesity and related diseases such as NAFLD (11). The gut microbiota's ability to treat NAFLD is extensively established (12). Meanwhile, some studies have found that NAFLD in mice can be reduced by adjusting the composition of intestinal flora, such as reducing the ratio of Firmicutes/Bacteroides, and improving the abundance of Akkermansia and Proteobacteria (13, 14). Based on this premise, previous reports have revealed that bioactive polysaccharides extracted from traditional Chinese medicine, particularly acidic polysaccharides, exhibit potential therapeutic effects against HFD-induced NAFLD (15). The observed effect is associated with the regulation of intestinal flora balance by polysaccharides, which can effectively mitigate blood lipid levels, reduce low-density lipoprotein (LDL) content, and inhibit lipid peroxidation in the liver (16).

*Crataegus pinnatifida* Bunge is a plant belonging to the Rosaceae family, which possesses both medicinal and edible value in its fruits and leaves (17). Researches have demonstrated that *Crataegus pinnatifida* fruit extract exhibits various beneficial biological functions, including antioxidizing, hypoglycemic, lipid control, intestinal flora regulation, immune modulation, and anti-tumor effects (18–20). Additionally, *Crataegus pinnatifida* leaf extract possessed lipid-lowering, anti-inflammatory, antioxidant, and antibacterial effects (21, 22). Thus, extracts, including the polysaccharides from different part of *Crataegus pinnatifida*, can be developed to drugs, health products and feed additives for the healthcare of human and animals.

In recent years, numerous reports have revealed the successful applications in the treatment of NAFLD. Through multiple synergistic mechanisms, polysaccharides have been proven to intervene in the development and occurrence of NAFLD by improving glucose and lipid metabolism, acting as antioxidants and anti-inflammatory agents, and regulating gut-liver interaction (23). These evidences indicated the potential value of polysaccharides on the intervene in the occurrence and progression of NAFLD.

*Crataegus pinnatifida* polysaccharide (CPP) was extracted from *Crataegus pinnatifida* fruit. In this study, we explored the therapeutic effects of CPP on C57/BL6J mice with NAFLD. We established the mouse model of NAFLD induced by a high-fat diet (HFD), determined the lipid content and liver function, studied liver histological sections, and characterized the genes and proteins related to lipid metabolism to evaluate the effectiveness of CPP treatment. The intestinal flora of mice was identified by 16S rRNA gene sequencing to investigate the way CPP can modulate the diversity of the microbiome. Our experiment studied the therapeutic effect of CPP on mice with NAFLD and found evidence for the development of healthy foods with anti-NAFLD properties.

## 2 Materials and methods

### 2.1 Extraction and purification of CPP

*Crataegus pinnatifida* Polysaccharide (CPP) was prepared by water extraction and alcohol precipitation (24). In brief, *Crataegus pinnatifida* fruit was processed with 90% ethanol (1:5, w/v) twice for 3 h to remove alcohol-soluble compounds, fat-soluble chemicals, and colors. The dry residue was extracted twice using hot water (1:30 w/v) for 2 h each. The two water extracts were mixed, and concentrated, then precipitated with 75% alcohol followed by centrifugation (8,000 r/min, 10 min) to acquire precipitation. The precipitation was dissolved with an adequate amount of water and finally centrifuged and freeze-dried to produce precipitation. DEAE cellular 52 (5.0 × 70.0 cm) and Sephadex G-100 columns were used to purify the CPP solution. Finally, CPP was produced through freeze-drying. The total sugar content of CPP was determined by phenol-sulfuric acid method (25). Briefly, Glucose standard solutions of different concentrations were prepared to plot a standard curve. *Crataegus pinnatifida* polysaccharide samples were then weighed to prepare the test solution. After treatment with the phenol-sulfuric acid method, the absorbance of the test solution was measured, and the polysaccharide content was calculated using the standard curve. The experiment was repeated three times in parallel.

### 2.2 Cellular lipid accumulation model and oil red O staining

The HepG2 cells were obtained from YaJi Biological (Shanghai, China) and cultured at 37°C in a humidified atmosphere of 5% CO_2_ in DMEM supplemented with 1% antibiotics and 10% FBS. Free fatty acid (FFA) solution was made by dissolving a 2:1 mixture of oleic and palmitic acids in DMEM containing 1% BSA, while heating the fluid to the proper temperature. The optimal concentrations for FFA molding and CPP treatment were determined using the CCK8 method. HepG2 cells were seeded in 6-well plates at a density of 1.25 × 10^5^ cells/well and incubated overnight. Subsequently, the HepG2 cells were treated with medium containing 1 mmol/L FFA along with CPP concentrations of 3, 4, or 5 mg/ml for a duration of 24 h. After cell homogenization, the supernatant was collected to determine aspartate aminotransferase (AST), alanine aminotransferase (ALT), total cholesterol (TC), and triglycerides (TG) levels using ELISA kits from Nanjing Jiancheng Bioengineering Institute, China, as per the provided instructions. The cells were stained with oil red O in accordance with the guidelines supplied by Beyotime Biotechnology (Shanghai, China). The stained cells were then extracted with isopropyl alcohol and their absorbance at 510 nm was measured.

### 2.3 Animals and treatment

Healthy adult male *C57*BL/6 mice (6 weeks) weighing 17–19 g were purchased from Comparative Medicine Centre of Yangzhou University. Animal studies were performed according to the guidelines of the Institutional Animal Care and Ethics Committee of Nanjing Agricultural (No. 20220918139).

As shown in **Figure 2A**, after 1 week of adaptive feeding, the mice were randomly divided into five groups: normal control group (CON), model control group (HFD), *Crataegus pinnatifida* polysaccharide low dose group (CPPL), *Crataegus pinnatifida* polysaccharide medium dose group (CPPM) and *Crataegus pinnatifida* polysaccharide high dose group (CPPH) (*n* = 6 each group). CON group was fed with ordinary diet, and other groups were fed with high-fat and high-cholesterol diet that contained 20% fat, 1% cholesterol, and 0.2% sodium cholate. After 16 weeks of feeding, mice in CPPL group, CPPM group, and CPPH group were administrated by CPP solution daily with the concentration of 100, 250, and 500 mg/kg, respectively. Mice in the CON group and HFD group received the same amount of water at the same moment. After 8 weeks of treatment, mice fasted 12 h before dissection, and then all mice were anesthetized with diethyl ether to harvest the blood. After that, mice were euthanized to collect the stool, epididymal fat, colon, and liver tissue for further analysis.

### 2.4 Measurement of biochemical indicators

The blood was centrifuged at a speed of 3,000 r/min for 10 min to obtain serum. An automated biochemical analyzer was then used to assess the amounts of TG, ALT, AST, TC, and low-density lipoprotein cholesterol (LDL-C) in the collected serum.

### 2.5 Histological analysis

Freshly extracted liver, epididymal fat, and colon were immediately immersed in 4% paraformaldehyde for 24 h. Then it was shipped to Powerful Biology Co., Ltd. (Wuhan, China) for H&E and Oil Red O staining. All of the above parts were examined using an optical microscope.

### 2.6 Quantitative real-time PCR analysis

A total RNA extraction was performed using Vazyme's RNA Isolator Total RNA Extraction Reagent (R401-01). RNA concentration and purity were detected by NanoDrop micro-spectrophotometer. The HiScript III 1st Strand cDNA Synthesis Kit (Vazyme, R312-02) was used to synthesize first-strand cDNA with oligo dT as the reverse transcription primer. Quantitative PCR (qPCR) was performed using a BIO-RAD real-time PCR system and the Hieff qPCR SYBR Green Master Mix (Yeasen, 11201ES08). β-actin was used as the control gene for normalization. The relative quantitative expression of genes in each group was analyzed by 2^−ΔΔCt^ and the relative expression was calculated. Table 1 displays the primer sequences.

**Table 1:** List of primers used for qRT-PCR.

**Gene**	**Forward primer**	**Reverse prime**
Human-CPT1A	ATTTTGCTGTCGGTCTTGGA	CTCTTGCTGCCTGAATGTGA
Human-PPARα	ACCACCATTCCCACAGACAG	CCAGGTTTGCGTAGAAGAGC
Human-β-actin	CATGTACGTTGCTATCCAGGC	CTCCTTAATGTCACGCACGAT
Mouse-PPARα	AACATCCAAGAGATTTCGCAATC	CCGTAAAGCCAAAGCTTCCA
Mouse-CPT1A	TGTTGGGTATGCTGTTCATGACA	GCGGCCTGGGTAGGAAGA
Mouse-FASN	CCGAGACACTCGTGGGCTA	CTTCAGCAGGACATTGATGCC
Mouse-ACC	GAGGGCTAGGTCTTTCTGGAAG	CCACAGTGAAATCTCGTTGAGA
Mouse-β-actin	TGTCCACCTTCCAGCAGATGT	AGCTCAGTAACAGTCCGCCTA

### 2.7 Western blot analysis

The liver was grinded for 30 min in the RIPA (Solarbio Life Sciences, Beijing). Then centrifuge the fissile at 12,000 rpm for 10 min. After adding the supernatant to the SDS-PAGE Sample Loading Buffer, the protein was denatured at 98°C for 15 min in a metal bath. The protein sample (60–70 μg) was separated using 7.5% SDS-PAGE and transferred to a PVDF membrane (Millipore, Billerica, MA, USA). The PVDF membrane was subjected to 5% skim milk powder at room temperature for 90 min. The primary and secondary antibodies were then incubated in succession. Antibodies were bought from Abmart (Abmart, Shanghai) and used as recommended by the manufacturer. The primary antibodies CPT1A (TD12004, dilution 1: 1,500), FASN (T56597, dilution 1: 1,500), ACC (T55575, dilution 1: 1,500), PPAR alpha (TA5301, dilution 1: 1,500), β-actin (P30002, dilution 1: 3,000), and Goat Anti-Rabbit Mouse IgG-HRP (M21003, dilution 1: 6,000) were used for target protein determination. Following the manufacturer's instructions, protein bands were seen using an enhanced chemiluminescence kit (Proteintech, PK10003).

### 2.8 Determination of fecal SCFAs concentrations

The contents of mice cecum were collected and the contents of short-chain fatty acids were analyzed by gas chromatography (GC) (Thermo Fisher Scientific, Waltham, MA, USA) (26).

### 2.9 16S rRNA sequencing analysis

Wuhan Software Biotechnology Co., LTD. (Wuhan, China) received fresh colon fecal samples that had been frozen with liquid nitrogen in order to do 16S rRNA sequencing analysis.

### 2.10 Statistical analysis

All data analysis was conducted by Ordinary one-way ANOVA Multiple comparisons in the GraphPad Prism software 9.0. Data are expressed as means ± SEM; Compare the mean of each column with the mean of HFD group.

## 3 Results

### 3.1 The total sugar content of CPP

Taking the standard concentration of glucose as the horizontal coordinate and the absorbance measured at 490 nm as the vertical coordinate, the standard curve was drawn, and the corresponding linear equation was calculated as *y* = 0.5656x + 0.0774, R2 = 0.9947. The average absorption value of Hawthorn polysaccharide at 490 nm was 0.531, and its polysaccharide content was 80.25%.

### 3.2 CPP improved lipid accumulation in HepG2 cells

**Figure 1:**
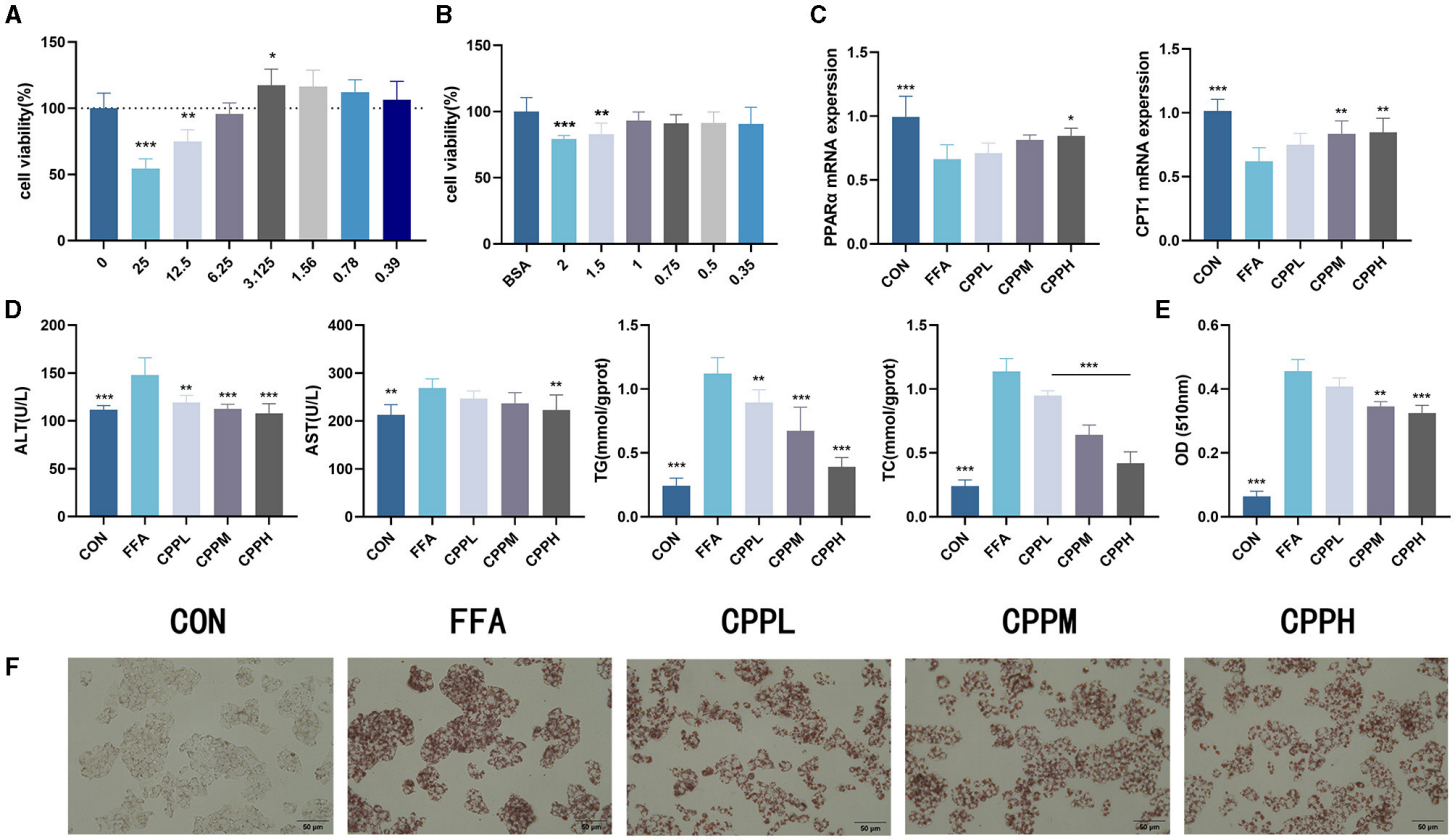
Effect of CPP on lipid accumulation in HepG2 cells. **(A)** Cell viability of HepG2 cells treated with CPP. **(B)** Cell viability of HepG2 cells treated with FFA. **(C)** PPARα and CPT1 expression levels. **(D)** Concentrations of AST, ALT, TG, TC in HepG2 cells. **(E)** Quantitative results of oil red O. **(F)** Oil red O staining of HepG2 cells (*n* = 6; *P < 0.05, **P < 0.01, ***P < 0.001 vs. HFD group).

To determine the incubation concentration of CPP, CCK8 assays were employed to assess the impact of CPP on cell viability. The findings revealed that CPP exhibited no cytotoxicity at concentrations below 6.25 mg/mL (Figure 1A). In order to investigate the effect of CPP on hepatocytes, a cellular lipid accumulation model was established using HepG2 cells, and CCK8 results demonstrated that 1 mM FFA was a suitable concentration for modeling purposes (Figure 1B). The measurement of AST, ALT, TC, and TG in HepG2 cells revealed that CPP lowered these levels in a dose-dependent manner (Figure 1D). As seen in Figures 1E, F, Oil red O staining and quantitative analysis of HepG2 cells revealed that CPP diminish FFA-induced lipid accumulation. In addition, CPP can boost mRNA expression of CPT1 and PPARα, important genes in fatty acid oxidation (Figure 1C).

### 3.3 CPP inhibits obesity and liver steatosis

**Figure 2:**
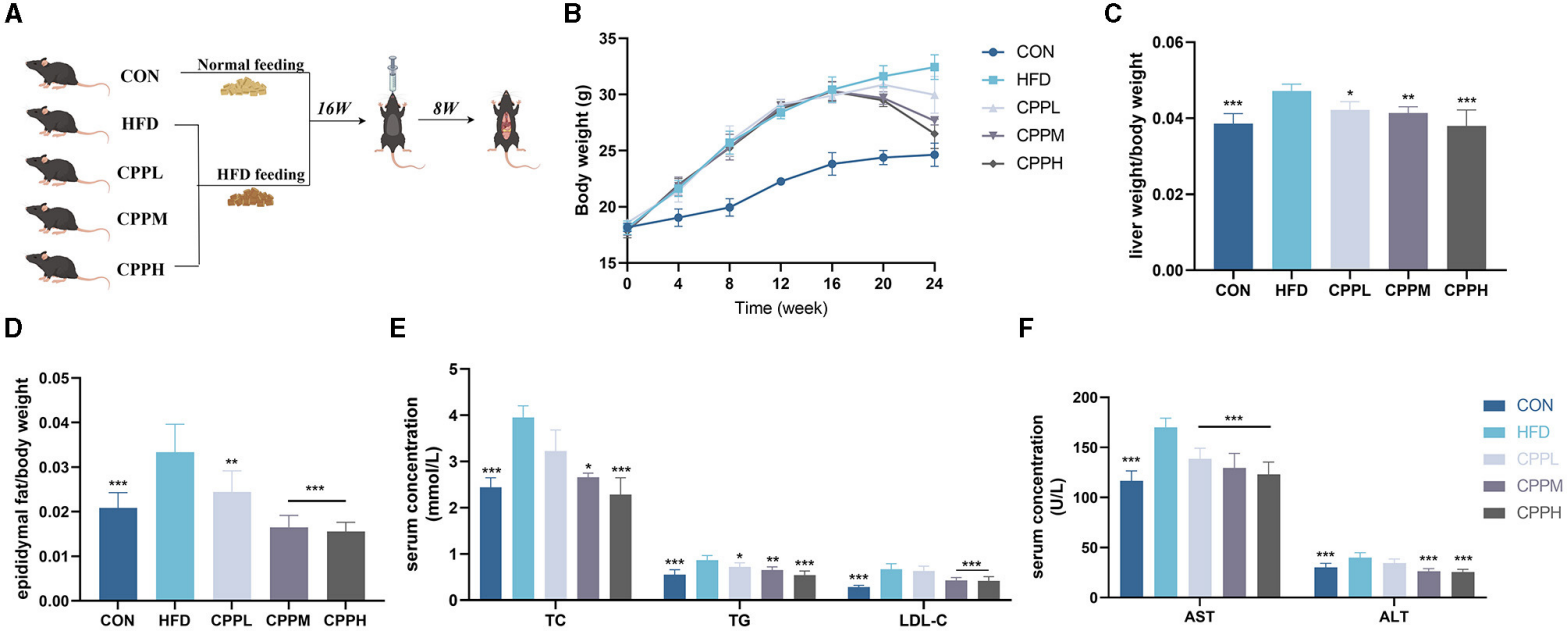
CPP improved the related parameters of NAFLD mice. **(A)** Schematic diagram of animal experiment process. **(B)** Body weight change curve. **(C)** Liver weight ratio. **(D)** Epididymal fat/body weight. **(E)** Concentrations of TC, TG and LDL-C in serum. **(F)** Concentrations of AST and ALT in serum (*n* = 6; *P < 0.05, **P < 0.01, ***P < 0.001 vs. HFD group).

As shown in Figure 2B, during the first 16 weeks, mice fed by HFD gained significantly more weight than mice in the control (CON) group. However, after the gavage of CPP, weight loss was observed in the HFD-fed mice. After 8 weeks of CPP intervention in NAFLD mice, the liver weight ratio in the CPPH group decreased significantly (Figure 2C), and CPP significantly reduced epididymal fat weight/body weight rate in a dose-dependent manner (Figure 2D). After CPP intervention, the contents of TC, TG, LDL-C, AST, and ALT in serum were significantly reduced (Figures 2E, F).

### 3.4 CPP alleviated liver steatosis and lipid accumulation

**Figure 3:**
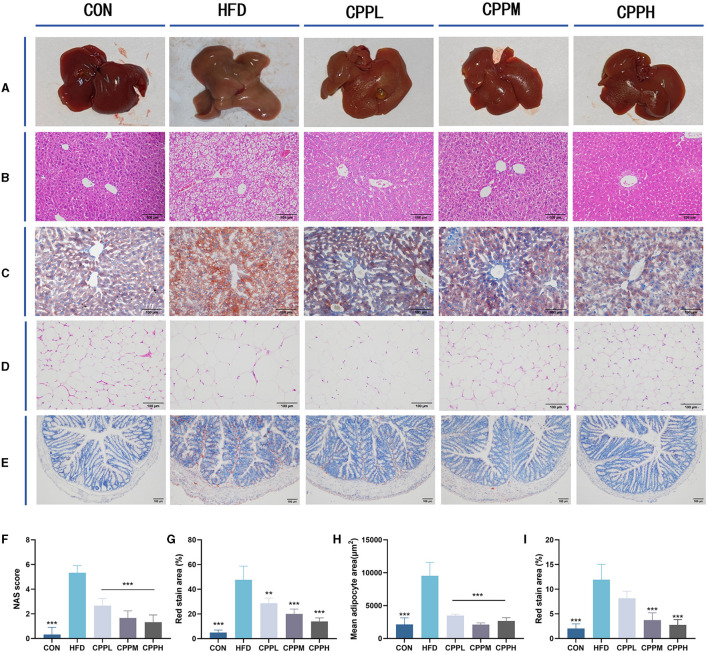
**(A)** Liver photograph. **(B)** H&E staining of liver. **(C)** Oil red O staining of liver. **(D)** H&E staining of epididymis fat. **(E)** Oil red O staining of colon tissue. **(F)** NAS score. **(G)** Oil red staining area of liver. **(H)** Mean adipocyte area. **(I)** Oil red staining area of colon (*n* = 6; **P < 0.01, ***P < 0.001 vs. HFD group).

The mice from the CON group had a smooth, sharply edged liver with a reddish-brown hue, as seen in Figure 3A. The livers from the HFD group were larger, with rounded margins and a brown-yellow tint. According to the Hematoxylin and Eosin staining (H&E) staining results, hepatocyte steatosis was observed in the HFD model group, and many vacuoles (fat droplets) in different sizes appeared in the cytoplasm (Figures 3B, C, F, G). In addition, the epididymal fat void area was significantly increased in the HFD group compared to the CON group (Figures 3D, H). However, after CPP treatment, the liver volume shrank and appeared reddish brown. Compared to the HFD group, the Oil Red O staining also showed a reduced oil droplet area of liver and colon through CPP administration (Figures 3E, I). These observations indicate that CPP can effectively inhibit the occurrence of fatty liver induced by HFD.

### 3.5 CPP regulated lipid metabolism

**Figure 4:**
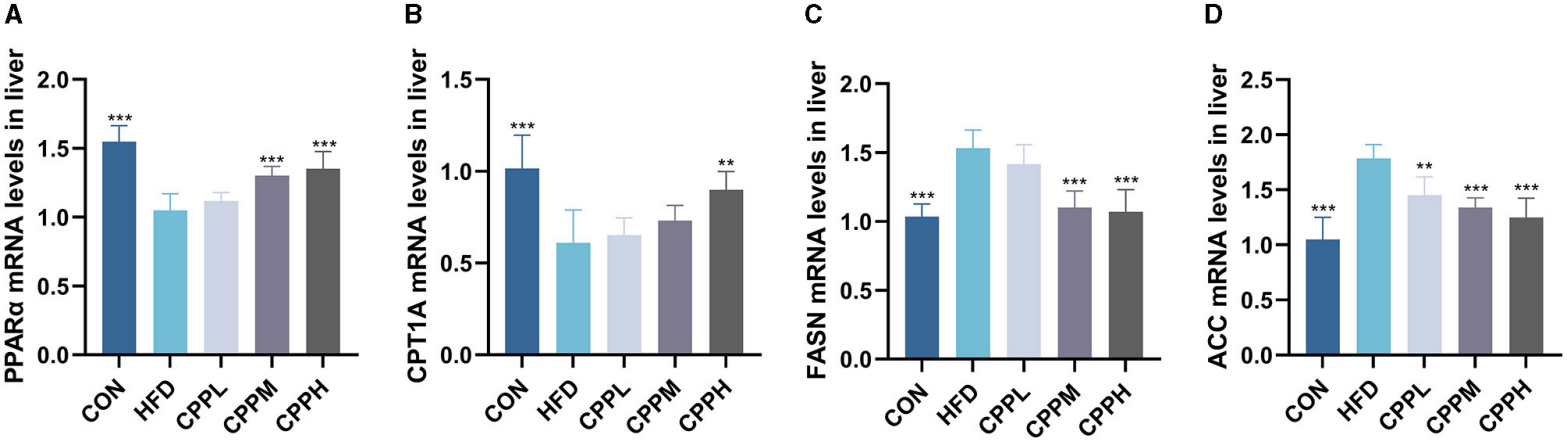
Expression of liver lipid metabolism-related genes: **(A)** PPARα. **(B)** CPT1A. **(C)** FASN. **(D)** ACC (*n* = 6; **P < 0.01, ***P < 0.001 vs. HFD group).

The key pathway of NAFLD was evaluated by mRNA expression. Figure 4 shows that, compared to the CON group, the FASN and ACC genes, which are deeply involved in fatty acid synthesis, showed pronounced elevated mRNA expression levels in the HFD group. Conversely, CPT1A and PPARα, which are key genes associated with fatty acid oxidation, have a significantly downregulated mRNA expression level. The CPP administration inhabit mRNA expression of FASN and ACC, while upregulated the CPT1A and PPARα gene expression, suggesting its ability to regulate the lipid metabolism.

**Figure 5:**
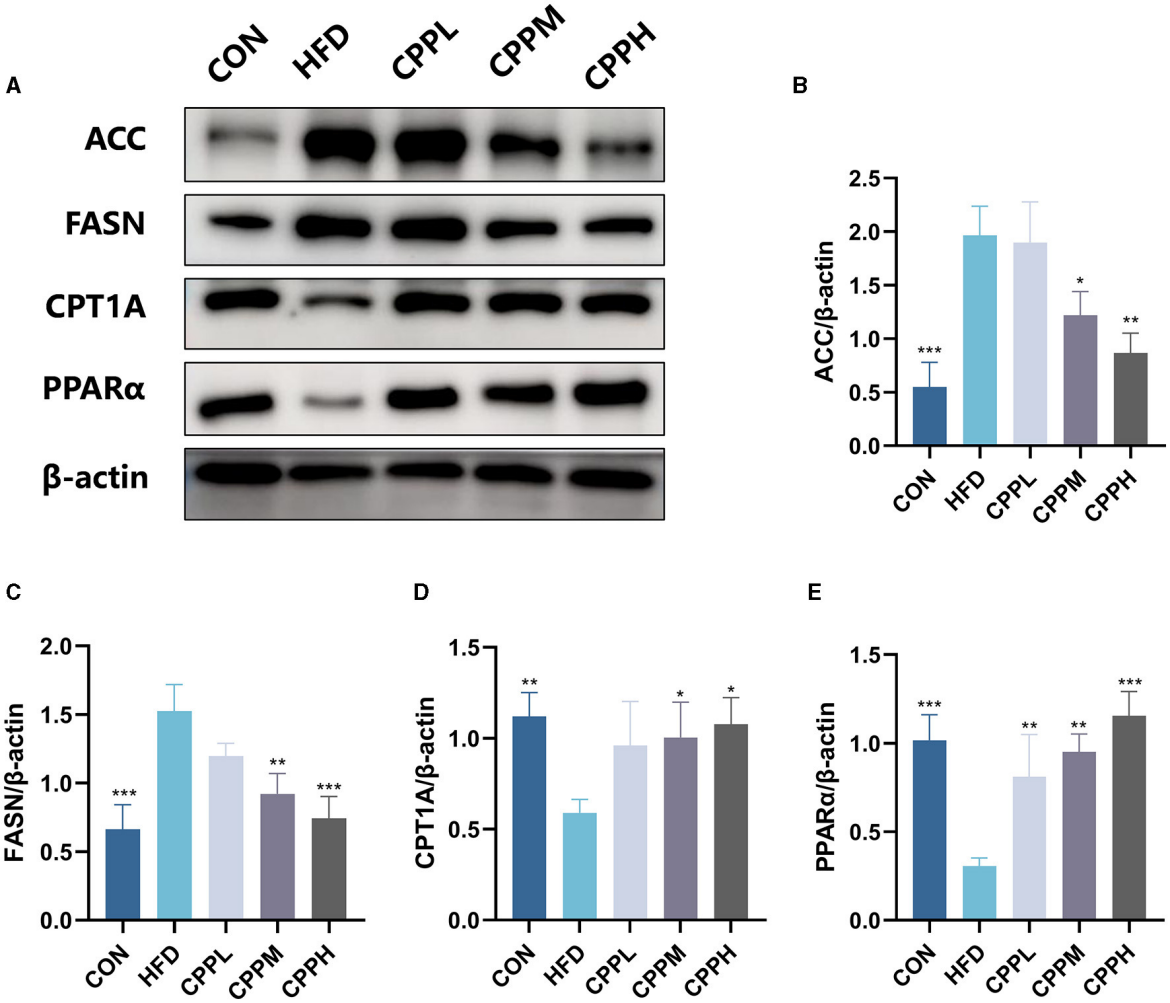
Expression level and statistical diagram of liver lipid metabolism-related proteins: **(A)** representative western blot image of ACC, FASN, CPT1A, PPARα, and β-actin. **(B)** Protein expression levels for ACC. **(C)** Protein expression levels for FASN. **(D)** Protein expression levels for CPT1A. **(E)** Protein expression levels for PPARα (*n* = 3; *P < 0.05, **P < 0.01, ***P < 0.001 vs. HFD group).

As shown in Figure 5, compared with the CON group, the relative expression levels of ACC and FASN were increased in HFD group, while the relative expression levels of CPT1A and PPARα were decreased. However, this phenomenon was reversed following CPP supplementation.

### 3.6 CPP regulates the composition of gut microbiota in NAFLD mice

**Figure 6:**
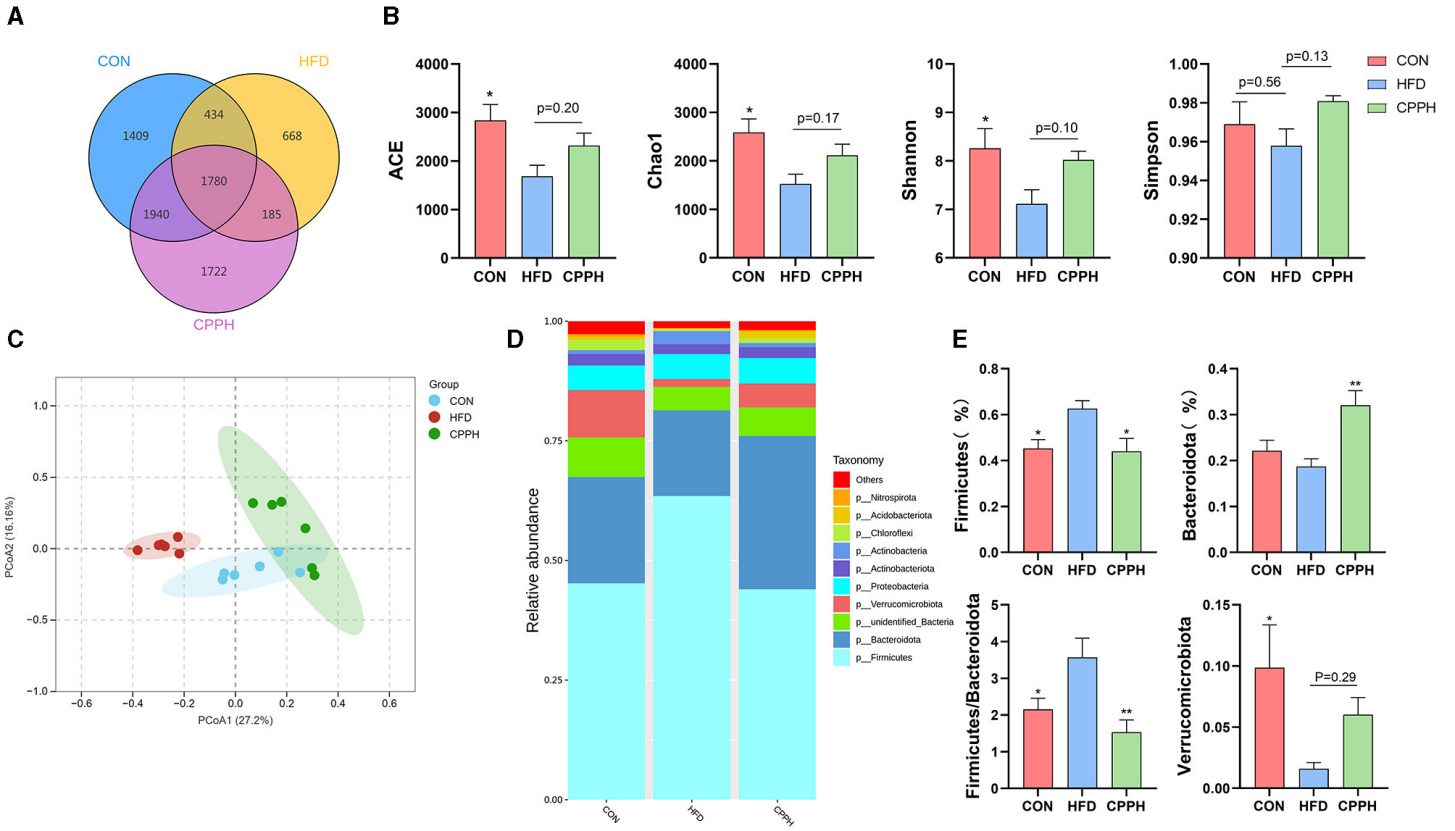
Effect of CPP on the microbiota of colon contents. **(A)** Venn diagram. **(B)** Alpha diversity. **(C)** PCA diversity. **(D)** Alterations in microbiota at phylum level. **(E)** Firmicutes abundances, Bacteroidota abundances, Firmicutes/Bacteroidota, and Verrucomicrobiota abundances (*n* = 6; *P < 0.05, **P < 0.01, vs. HFD group).

**Figure 7:**
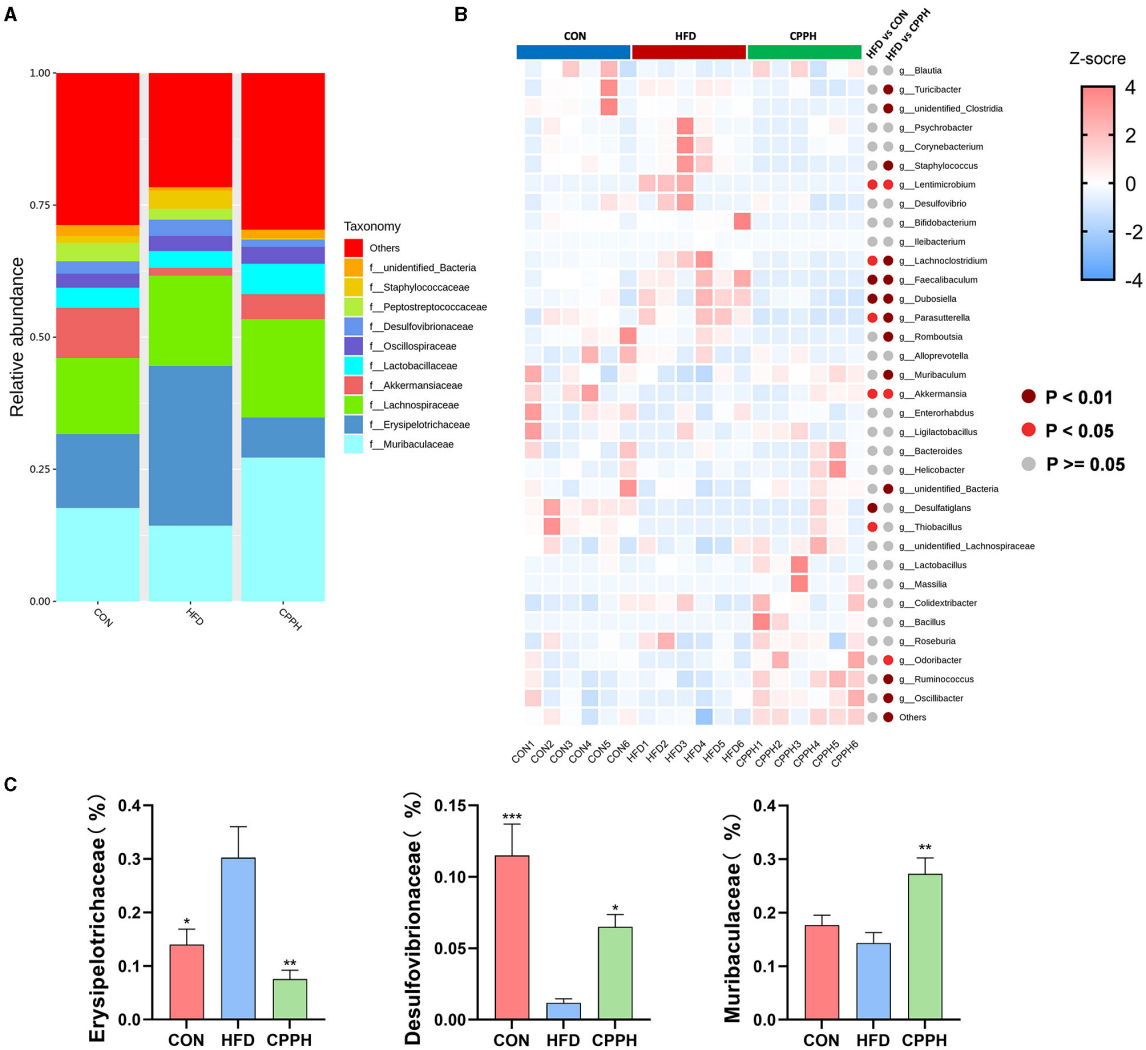
**(A)** Alterations in microbiota at family level. **(B)** Heatmap of the top35 altered by HFD responding to cpp supplementation in genus level. **(C)** Erysipelotrichaceae abundances, Desulfovibrionaceae abundances, Muribaculaceae abundances, Turicibacter abundances (*n* = 6; *P < 0.05, **P < 0.01, ***P < 0.001 vs. HFD group).

Based on the above results, the CPPH group showed the best therapeutic effect against NAFLD. Therefore, samples of this group were selected for 16s analysis. As depicted in Figures 6A, B, the Venn diagram illustrates that 1,780 OTUs are shared across all groups, while certain OTUs are unique to specific groups (1,409 for CON, 668 for HFD, and 1,722 for CPPH). The alpha diversity index includes the Chao1 index, ACE index, Shannon-Channon index, and Simpson index. The results revealed that the alpha diversity index was higher in the CPPH group compared to the HFD group. As depicted in Figure 6C, there is an overlap between the CON group and CPPH group, while the samples from the HFD group exhibit relatively large distances, indicating significant alterations in overall community composition among these three groups. To further investigate differences in mice intestinal flora structure across each group, we conducted an analysis based on gate-level classification. By comparing the results at the gate level (Figure 6D), it is obvious that the intestinal microflora of the mice across each experimental group was dominated by Firmicutes and Bacteroidetes. In contrast to the CON group, the proportion of firmicutes in the intestinal flora of the HFD group is the highest, as seen in Figure 6E, which was reduced by CPP treatment. Considering the significance of Firmicutes/Bacteroidetes (F/B) values in metabolic syndrome, we analyzed the F/B rate in the intestinal microbiota structure of mice in each group. Based on the results, the F/B rate of the HFD group was significantly higher than that of the CON group, which can be lowered by CPP treatment. In addition, mice fed a high-fat diet had a significant decrease in Verrucomicrobiota abundance (Figure 6E). Further comparisons among the results at the family level were performed (Figures 7A, C). Compared to the CON group, the proportion of Erysipelotrichaceae in the HFD group's intestinal flora increased significantly, while the proportion of Desulfovibrionaceae decreased. However, this phenomenon was reversed following CPP supplementation. Importantly, a considerably change of the proportion of Muribaculaceae can be observed in the intestinal microbiota of NAFLD mice treated by CPP.

**Figure 8:**
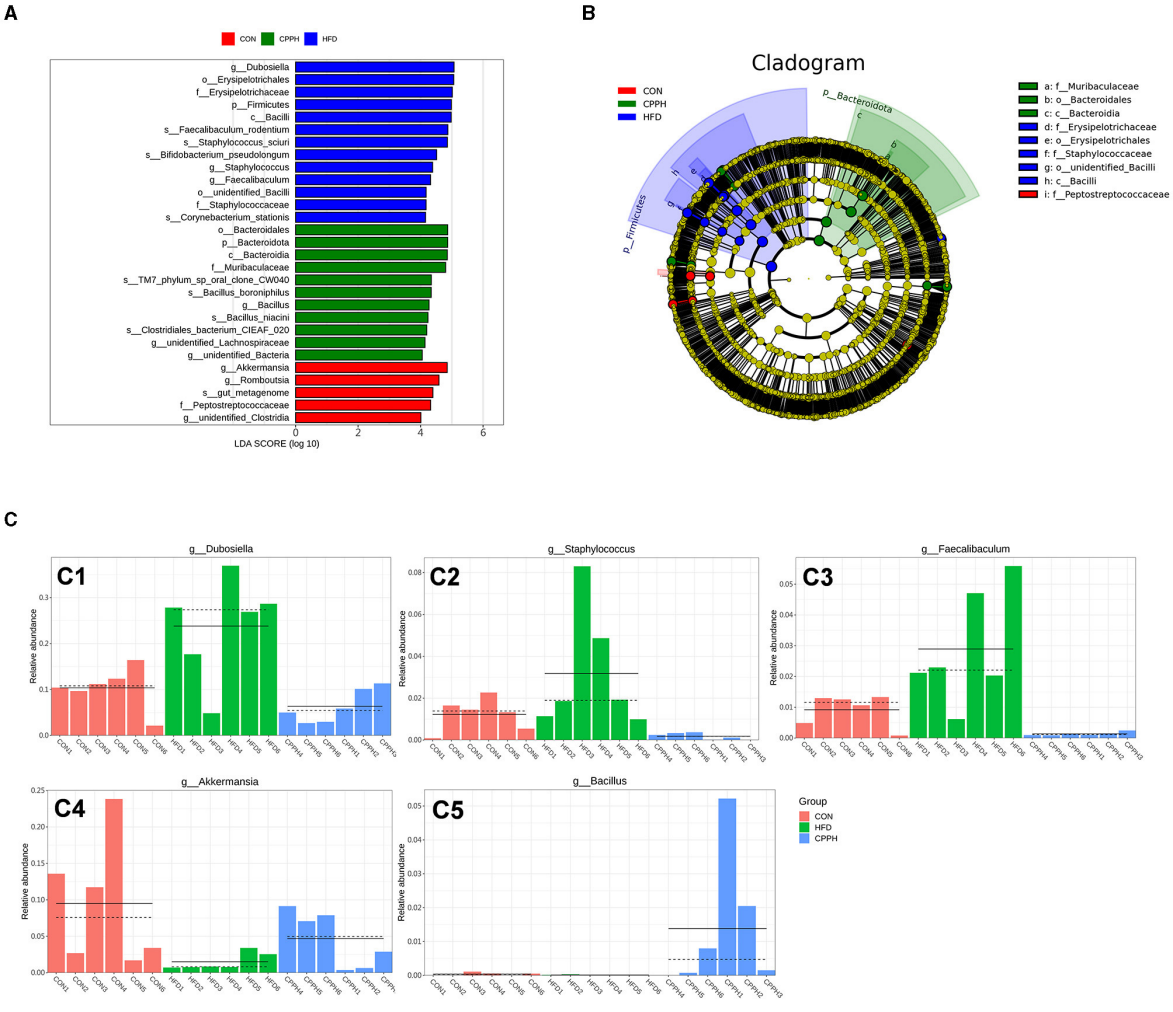
**(A)** LDA value distribution histogram based on OUT, LDA ≥ 4. **(B)** Evolutionary branching diagram based on OUT. **(C)** The relative abundance of key genera. C1: Dubosiella, C2: Staphylococcus, C3: Faecalibaculum, C4: Akkermansia, C5: Bacillus (*n* = 6).

To evaluate the influence of CPP supplementation on major bacterial genera, a statistical analysis was performed on the top 35 microorganisms at the genus level (Figure 7B). The results revealed significant changes in 8 bacterial genera in the HFD group compared to CON. Mice administered with CPP exhibited significant alterations in 16 bacterial genera compared to the HFD group. Notably, CPP reversed changes in six bacterial genera caused by HFD. Based on the evolutionary cladistic diagram and LEfSe diagram of ASV (LDA ≥ 4), we identified five key bacterial genera among the top 35 abundant ones that were jointly affected by both HFD and CPP (Figures 8A, B). These specific bacterial genera may be potential contributors to CPP's beneficial effects on treating NAFLD. Our experiment revealed that HFD led to an enriched Dubosiella, Staphylococcus, and Faecalibaculum distribution (Figure 8 C1–C3), while depleting Akkermansia (Figure 8 C4). However, supplementation with CPP could effectively reverse these alterations. Additionally, the Bacillus in NAFLD mice became more prevalent after CPP administration. These changes in bacterial genera may reveal the mechanisms underlying the beneficial effects of CPP on NAFLD, namely CPP supplementation not only restores HFD-induced dysbiosis but also promotes probiotic enrichment.

### 3.7 Regulatory effect of CPP on SCFAs in NAFLD mice

**Figure 9:**
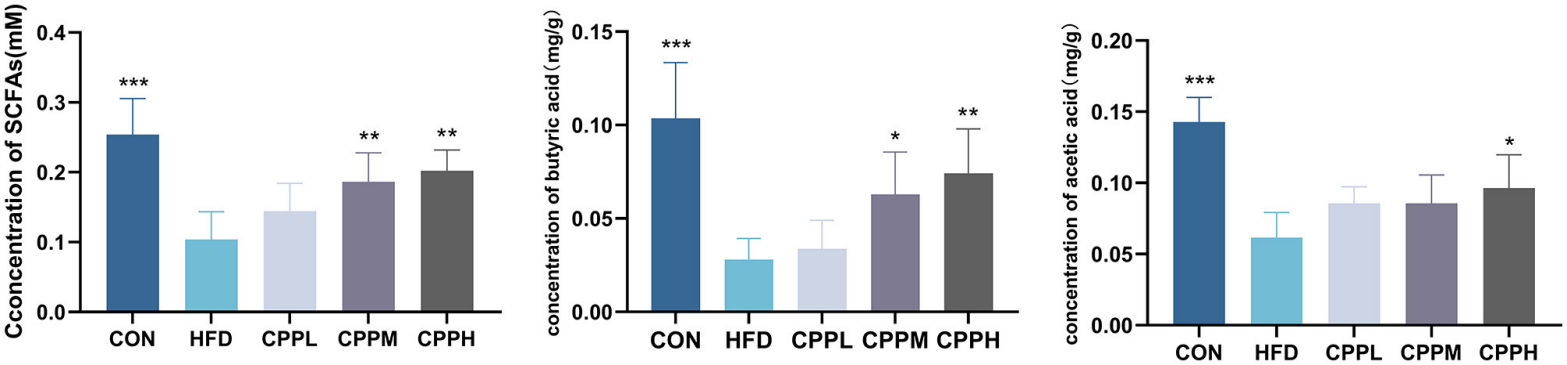
Total SCFA, butyric acid and acetic acid concentrations in the feces of mice (*n* = 6; *P < 0.05, **P < 0.01, ***P < 0.001 vs. HFD group).

SCFAs are a group of metabolites produced by gut bacteria during the fermentation of dietary fiber, which play an indispensable role in maintaining intestinal health. Our study found that the CPPH group showed significant increases in the amount of total SCFA, butyric acid, and acetic acid in feces compared to the CON group, suggesting that CPP can promote the activity of gut bacteria (Figure 9).

## 4 Discussion

Research on polysaccharides and NAFLD has advanced significantly in recent years, with multiple studies demonstrating that polysaccharides can successfully impede the onset and progression of NAFLD through a variety of mechanisms (27–30), such as enhancing glucose and lipid metabolism, exerting antioxidant and anti-inflammatory actions, and modulating the gut-liver axis (31). Polysaccharide, as an essential natural product, is drawing increasing attention due to its unique benefits, such as low toxicity, high safety, multi-targeting ability, and various mechanism of actions in different scenarios. In this study, we extracted *Crataegus pinnatifida* polysaccharide from *Crataegus pinnatifida* fruit, established an FFA-induced lipid accumulation model of HepG2 cells, and discovered that CPP may inhibit lipid accumulation in HepG2 cells by controlling fatty acid oxidation. Subsequently, we induced a mice NAFLD model with HFD and then intervened with different concentrations of CPP. After 2 months of CPP administration, the liver weight ratio and epididymal fat proportion of HFD mice were significantly decreased, together with the levels of TG, TC, LDL-C, AST, and ALT in serum. The relationship between obesity and hyperlipemia has been demonstrated and characterized by elevated concentrations of serum total cholesterol (TC) and triglycerides (TG) (32). CPP can decline the content of TC and TG in the serum of NAFLD mice in a dose-dependent manner. In addition, AST, ALT, and LDL-C levels were closely associated with NAFLD (33, 34). Similarly, histological examination showed that CPP can dose-dependently reduce liver injury and hepatic lipid accumulation induced by HFD in mice. These findings suggested that CPP possessed the ability to suppress excessive lipid accumulation.

Enhanced *de novo* adipose synthesis in the liver is considered a distinctive characteristic of individuals with NAFLD, contributing to adverse metabolic outcomes and exacerbating steatosis (35). The acetyl-CoA carboxylase (ACC) and fatty acid synthase (FASN) are pivotal regulators of NAFLD and hold promise as therapeutic targets for NAFLD (36, 37). Relying on ATP for energy supply, ACC serves as a rate-limiting enzyme in fat synthesis. The acetylated form of malonyl-CoA inhibits the activity of Carnitine cotransferase 1A (CPT1A) through feedback regulation, thereby impeding fatty acid oxidation (38). On the other hand, characterized by increased hepatic fatty acid production, fatty acid synthesis is a crucial pathway in the development of NAFLD. As a core regulatory factor, FASN facilitates the hepatic *de novo* lipogenesis by catalyzing the conversion of malonyl-CoA and ACC into palmitate, which subsequently undergoes elongation, desaturation, and esterification, ultimately leading to triglyceride accumulation in the liver (39). Our data indicate that CPP can effectively suppress both the mRNA and protein levels of FASN and ACC activity. The peroxisome proliferator-activated receptor α (PPARα) signaling pathway is crucial for lipid metabolism in the liver (40). CPT1A and PPARα play crucial roles in the oxidative metabolism of fatty acids (41). Upon activation, PPARα acts as a transcription factor to regulate the expression of target genes involved in lipid metabolism, such as fatty acid β oxidation (42). Activated PPARα also promotes the expression of CPT1A (43). With excessive liver lipid accumulation, down-regulation of CPT1A expression leads to an imbalance between β-oxidation and steatosis, resulting in lipid per-oxidation (44). Our results demonstrate that CPP elevates the mRNA expression of PPARα and CPT1A, hence promoting lipid metabolism activity in the livers of NAFLD mice.

NAFLD is the most common among chronic liver diseases. Intestinal dysbiosis, oxidative stress, and low-grade inflammation may be caused by dietary, pharmaceutical, or environmental factors, eventually leading to NAFLD (31). Polysaccharides have been shown to improve NAFLD by modulating gut microbiota ecology. Shu et al. found that *Auricularia auricula* polysaccharides (AAPs) could regulate gut dysbiosis in NAFLD mice by enriching *Bacillus odoricus*, Lactobacillus, Doreia, and bifidobacteria (29). Similarly, Zhu et al. demonstrated that *Ostrea rivularis* polysaccharides (ORP) could reduce Firmicutes and Proteobacteria abundance as well as the F/B ratio in NAFLD mice (45). These results suggest that ORP can modulate gut microbiota composition in NAFLD mice while enhancing intestinal barrier function and reducing permeability to delay disease progression and occurrence. Studies have indicated a potential association between Bacteroidetes and Firmicutes alterations with NAFLD (46). Firmicutes and Bacteroidetes are two major phyla of mammalian gut microbiota, and an increase in F/B ratio is generally considered the indication of HFD-induced microbial imbalance (47). Our 16sRNA analysis showed that CPP changed the composition of the gut microbiome in NAFLD mice, reducing the value of F/B and reversing the imbalance of the gut microbiome, which is in accordance with previous studies. Verrucomicrobiota was consistently associated with a reduced abundance of pathogenic microbes and improved host metabolism under high-fat diet conditions (48).

Many studies have found that Erysipelotrichaceae is associated with dyslipidemia phenotype, and an increase of Erysipelotrichaceae has been observed in mice fed HFD (49–51). At the family level, the proportion of Erysipelotrichaceae bacteria in the intestinal flora of NAFLD mice increased as a result of NAFLD induction. After administration of a high dose of CPP, the abundance of Erysipelotrichaceae bacteria decreased, compared to the HFD group. Muribaculaceae, as a beneficial bacterium involved in butyrate production, has demonstrated potential effects on host health specifically related to obesity, metabolic disorders, and other gastrointestinal diseases (52–54). The proportion of Muribaculaceae in the gut microbiota of CPP-treated NAFLD mice elevated considerably. At the genus level, we integrated cladistic and LEfSe diagrams of ASV (LDA ≥ 4) and observed that CPP can mitigate the reduction in Akkermansia abundance induced by HFD. which is widely recognized as a key beneficial microbial component that protects against Western diet-induced atherosclerosis in mice (55), and a potential probiotic for the treatment of NAFLD (56). Jiang et al. found that oral TLP promoted the growth of characteristic strains Ligilactobacillus and Akkermansia in HFD-fed rats (57), thereby alleviating the development of NAFLD, which is consistent with our results. Several studies have proposed that SCFAs might influence the progression of NAFLD through their regulation of hepatic lipid metabolism and suppression of inflammatory responses. For example, DQW treatment promoted SCFAs production in NAFLD mice and subsequently improved intestinal barrier integrity and inflammation (58). In addition to serving as fuel for the body, SCFAs produced by polysaccharide metabolism inside the gut microbiota also act as signaling molecules in lipid metabolism and inflammatory response (10). Apart from regulating short-chain fatty acid synthesis (SCFA), polysaccharides can also improve NAFLD. Researchers found that *Poria* water-insoluble polysaccharide (WIP) significantly increased the relative abundance of bacteria that produce butyric acid in NAFLD mice; in the meantime, butyrate upregulated tight binding protein and mucosal integrity protein (muc5) expression (59). Furthermore, butyrate has the potential to induce protective transfer of the intestinal microbiome, restore the intestinal barrier in mice, and effectively prevent NASH-related liver injury induced by MCD diet (60). Additionally, *D. versgaris*, enriched in *Astragalus* polysaccharides (APS), may effectively mitigate hepatic steatosis through acetic acid production and regulation of lipid metabolism in mouse liver (61). Our study revealed a significant increase in total SCFA content as well as butyric acid and acetic acid levels in feces following CPP intervention, suggesting that CPP could ameliorate the progression of NAFLD by regulating imbalances within intestinal flora and SCFAs. In conclusion, CPP has the ability to regulate lipid-related pathways via regulating gut microbiota and short-chain fatty acids, consequently slowing the progression of NAFLD in mice.

## Data availability statement

The original contributions presented in the study are publicly available. This data can be found here: https://www.ncbi.nlm.nih.gov/bioproject/; PRJNA1075301.

## Ethics statement

The animal study was approved by Institutional Animal Care and Ethics Committee of Nanjing Agricultural (No. 20220918139). The study was conducted in accordance with the local legislation and institutional requirements.

## Author contributions

PH: Conceptualization, Visualization, Writing – original draft, Writing – review & editing. XY: Visualization, Writing – original draft. WY: Validation, Writing – review & editing. XW: Validation, Writing – original draft. YL: Validation, Writing – original draft. MZ: Validation, Writing – original draft. YYu: Methodology, Writing – original draft. SC: Software, Validation, Writing – original draft. YYua: Methodology, Writing – original draft. XQ: Software, Writing – original draft. ZX: Methodology, Writing – original draft. JZ: Investigation, Writing – original draft. WZ: Writing – review & editing. YZ: Methodology, Writing – original draft. CS: Project administration, Writing – original draft. QX: Methodology, Writing – original draft. SQ: Formal analysis, Writing – original draft. YW: Conceptualization, Data curation, Funding acquisition, Methodology, Project administration, Supervision, Writing – review & editing. XS: Funding acquisition, Writing – review & editing. KW: Funding acquisition, Writing – review & editing.
